# Development and expert evaluation of a culturally adapted large language model for Malay clinical history-taking simulation: mixed methods study

**DOI:** 10.3389/fmed.2026.1895321

**Published:** 2026-09-30

**Authors:** Noor Akmal Shareela Ismail, Ahmad Hathim Ahmad Azman, Azizah Zafira, Muhammad Faris Ahmad Faiz, Syahrul Sazliyana Shahahir, Ruslinda Mustafar, Chairozila Mohd Shamsuddin, Daing Zairi Ma’arof, Shahnorbanun Sahran

**Affiliations:** 1 Faculty of Medicine, Universiti Kebangsaan Malaysia, Kuala Lumpur, Malaysia; 2 Centre for Artificial Intelligence Technology (CAIT), Faculty of Information Technology, Universiti Kebangsaan Malaysia, Bangi, Selangor, Malaysia; 3 Faculty of Liberal Science, Universiti Kebangsaan Malaysia, Bangi, Selangor, Malaysia

**Keywords:** clinical history-taking, cultural adaptation, fine-tuning, large language models, Malaysia, medical education, simulation-based learning

## Abstract

**Background:**

Clinical history-taking is a core medical competency, but conventional teaching may be limited by patient availability, variable clinical exposure, and restricted opportunities for repeated practice. Large language models (LLMs) may support simulated practice; however, their clinical, linguistic, and cultural suitability requires careful evaluation in multilingual settings.

**Objective:**

This study aimed to develop and expert-evaluate a culturally and linguistically adapted English–Malay clinical dialogue corpus and to descriptively benchmark a fine-tuned LLM framework for clinical history-taking simulation in Malaysian undergraduate medical education.

**Methods:**

A multiphase mixed-methods development and evaluation study was conducted across nine internal medicine systems. Of 63,250 AI-generated dialogue entries, each comprising a simulated doctor inquiry and corresponding patient response, 59,184 (93.6%) underwent consultant-level subject matter expert review using an author-developed holistic 5-point rubric informed by clinical relevance, accuracy, clarity and coherence, completeness, and human-likeness. Free-text comments were analysed inductively. After SME evaluation and clinical-quality and mechanical filtering, 58,886 entries across 2,497 simulated patient profiles were retained. The profiles were partitioned into 1,997 training profiles, 250 validation profiles, and 250 held-out test profiles. Four model configurations were descriptively benchmarked using the same held-out profiles and an automated large language model judge. This benchmark was intended for internal model comparison and did not constitute independent external or human clinical validation of the post-fine-tuning model outputs.

**Results:**

Mean subject matter expert scores were 4.52/5 for simulated doctor inquiries and 4.58/5 for simulated patient responses. Qualitative analysis identified recurring limitations involving question structure, incomplete probing, repetition, linguistic accuracy, over-explanation, and simulated-patient realism. In descriptive benchmarking, the fine-tuned model achieved the highest observed mean score among the four configurations evaluated.

**Conclusions:**

Developing a consultant-reviewed, culturally adapted English–Malay clinical dialogue corpus and using it to fine-tune an LLM for simulated history-taking was feasible. The fine-tuned model achieved the highest observed score in the internal automated benchmark, but this finding does not establish statistical, clinical, or educational superiority. The post-fine-tuning model outputs were not independently evaluated by clinicians in the present study. The framework may offer a scalable, contextually relevant supplement to clinical communication training, but blinded human evaluation of post-fine-tuning outputs, targeted safety assessment, external validation, and learner-outcome evaluation are required before routine student-facing implementation.

## Introduction

Clinical history-taking is a fundamental component of medical practice and a core competency in undergraduate medical education. It enables learners to obtain clinically relevant information, establish therapeutic rapport, and generate diagnostic hypotheses that guide subsequent clinical decision-making. A structured history-taking approach, incorporating domains such as the presenting complaint, history of presenting illness, past medical history, medication history, family history, and social history, provides a systematic framework for organising and synthesising patient information. This process supports the development of clinical reasoning by guiding learners through progressive information gathering and hypothesis-driven interpretation (1, 2).

Beyond its contribution to clinical reasoning, structured history-taking can support self-directed learning by helping students identify gaps in their clinical knowledge and questioning strategies (3). In assessment settings, structured approaches allow clinical performance to be evaluated more consistently through standardised rubrics, checklists, Objective Structured Clinical Examinations (OSCEs), and other assessment formats (1, 4, 5). These approaches are aligned with contemporary educational paradigms, including programmatic assessment and competency-based medical education, which emphasise longitudinal competency development, continuous feedback, and learner progression (6, 7).

Despite their educational value, conventional approaches to teaching history-taking, including didactic teaching, peer role-play, bedside teaching, and standardised patient encounters, can be constrained by patient availability, logistical and staffing requirements, variability in clinical exposure, and limited opportunities for repeated practice and timely formative feedback (8, 9). These constraints may reduce learners' exposure to diverse clinical presentations and their ability to practise challenging consultations in a psychologically safe and reproducible environment. Consequently, there has been increasing interest in digital and artificial intelligence (AI)-supported technologies that can provide scalable, accessible, and flexible learning environments within medical education (10, 11).

The LLM-based simulation framework examined in this study was designed to provide learners with repeated opportunities to practise clinical history-taking through a digital conversational environment. This design is consistent with the broader role of mobile and ubiquitous health technologies in extending access to flexible, technology-supported training (10, 11). In resource-constrained educational settings, such simulation could support distributed practice beyond the physical ward, clinical skills laboratory, or scheduled teaching session. However, its educational value depends on the clinical accuracy, linguistic appropriateness, and authenticity of the simulated interactions (12, 13).

Earlier conversational agents in health care were commonly implemented as text-based chatbots or digital assistants, with applications including treatment monitoring, patient education, screening, follow-up, health service support, and data collection (11, 14). Although these systems demonstrated the potential of conversational interfaces in health care, evidence regarding their safety, effectiveness, acceptability, and adaptability to different clinical and cultural contexts remained limited (11, 14).

Recent advances in large language models (LLMs) have enabled more contextually coherent, responsive, and human-like interactions. Emerging evidence suggests that LLMs can perform at or near medical learner benchmarks in selected knowledge-based assessments and clinical question-answering tasks (15–17). Nevertheless, performance in these settings does not necessarily establish suitability for simulated patient interaction. Concerns remain regarding factual inaccuracies, hallucinations, response inconsistency, poor calibration, and limited clinical reliability (18–20).

Systematic reviews have demonstrated growing interest in LLM applications across medical specialties and patient care while highlighting limited evaluation standardisation, insufficient transparency, bias, incorrect outputs, non-reproducibility, and variable performance across tasks (19, 20). These limitations may be particularly important when LLMs are used to simulate patients because unrealistic or clinically inappropriate responses could reinforce ineffective questioning strategies or introduce misconceptions. Furthermore, many general-purpose LLMs are developed predominantly using high-resource language data and may inadequately represent the linguistic and sociocultural characteristics of multilingual health care settings (21).

This concern is particularly relevant in Malaysia, where Malay and English coexist in medical education and everyday clinical communication (21, 22). Medical education may be conducted largely in English, while patients frequently describe their symptoms, personal concerns, and illness experiences in Malay or a combination of both languages. During a single consultation, a speaker may describe symptoms using colloquial Malay while retaining English clinical, anatomical, or medication-related terms. Language choice may also vary according to the patient's educational background, cultural identity, familiarity with medical terminology, and relationship with the clinician. These patterns can influence how symptoms, emotional concerns, uncertainty, and culturally shaped understandings of illness are expressed and interpreted.

Bilingual capability and code-switching are related but distinct concepts. In this study, bilingual capability refers to the availability of dialogue content in both English and Malay, whether presented separately or as parallel-language versions. Code-switching refers to alternation between English and Malay within an individual utterance or across conversational turns. Therefore, producing separate English and Malay versions of the same interaction demonstrates bilingual capability but does not, by itself, demonstrate an ability to interpret or generate natural code-switching. The present framework was developed using parallel English–Malay content; code-switched exchanges were not separately labelled or evaluated. Accordingly, the framework's ability to reproduce or interpret naturally occurring code-switching was not established in this study.

Recent initiatives have sought to address linguistic and contextual limitations through the development of Malay health corpora, resource-efficient Malay or Nusantara language models, and LLMs adapted to the Malaysian context (23–25). However, their integration into clinically evaluated educational applications remains limited. Preliminary research involving generative AI virtual patients has shown that LLMs can produce plausible responses for history-taking practice but may also generate implausible information, hallucinations, incomplete responses, reduced linguistic fluency, and unnatural patient behaviour (26). These findings indicate that technical language capability alone is insufficient; clinical curation, expert review, and contextual adaptation are also required.

Culturally and linguistically adapted LLM-based systems may therefore provide more contextually appropriate support for AI-assisted medical education. However, evidence remains limited regarding the development and evaluation of clinically reviewed English–Malay conversational datasets for undergraduate history-taking practice. To address this gap, the present study aimed to develop and evaluate a culturally and linguistically adapted English–Malay LLM framework for simulated clinical history-taking within Malaysian undergraduate medical education. By incorporating a structured history-taking framework, clinically curated conversational data, consultant-level expert review, and local linguistic adaptations, the study sought to bridge the gap between the technical capabilities of LLMs and their pedagogically appropriate application in a multilingual medical education setting.

## Methods

### Study design

This study used a sequential multiphase mixed-methods design to develop, evaluate, and descriptively benchmark a culturally and linguistically adapted large language model (LLM) framework for English–Malay clinical history-taking simulation. The framework was developed within the Year 3 Internal Medicine educational context at Universiti Kebangsaan Malaysia (UKM).

The study comprised four phases: dataset generation and subject matter expert (SME) evaluation; dataset preparation; parameter-efficient model fine-tuning; and internal descriptive model benchmarking. SME evaluation in Phase 1 assessed the AI-generated development corpus. Phase 4 subsequently compared outputs produced by four model configurations using an automated LLM judge and was intended as an internal model-selection benchmark rather than clinical validation of the final fine-tuned model. The overall workflow is presented in Figure 1.

**Figure 1:**
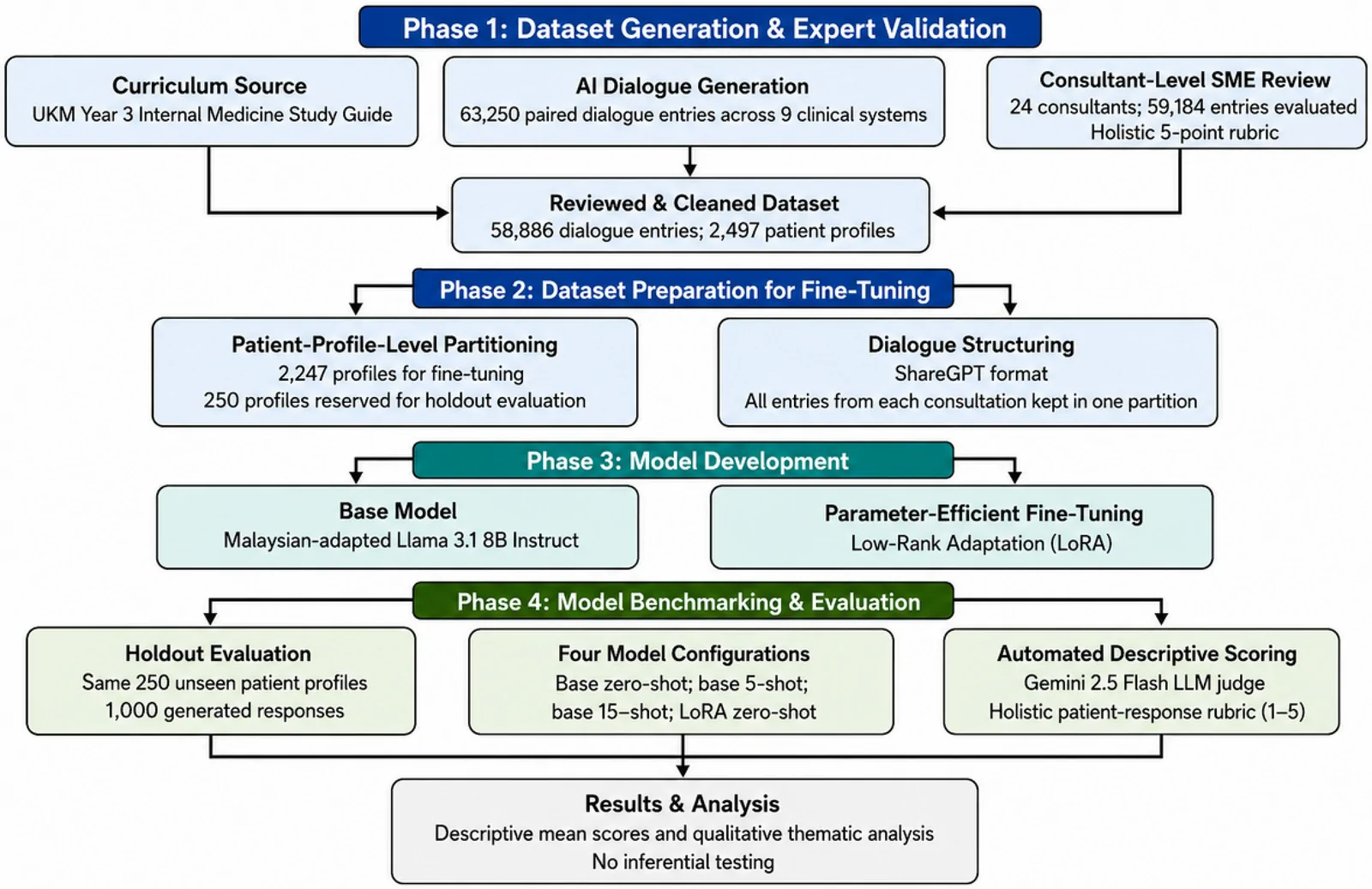
Multiphase study workflow. The workflow comprised AI-assisted dialogue generation, consultant-level SME evaluation, clinical-quality and mechanical filtering, patient-profile-level training–validation–test partitioning, 4-bit QLoRA fine-tuning, and internal descriptive benchmarking.

### Phase 1: dataset generation and SME validation

#### Structured history-taking framework

A structured history-taking framework was generated specifically for this study through an LLM-assisted process under the guidance of clinical educators. It was not adopted from a textbook or previous study. Its clinical scope was aligned with the Universiti Kebangsaan Malaysia (UKM) Year 3 Internal Medicine curriculum and reviewed for consistency with standard clinical history-taking guidance (27, 28).

Clinical history-taking was organised into predefined domains comprising the presenting complaint, history of presenting illness, past medical history, past surgical history, medication history, allergy history, family history, social history, and review of systems. Each domain was divided into clinically relevant subcomponents to guide the generation of contextually appropriate simulated doctor inquiries and patient responses.

The framework incorporated progressive disclosure, whereby clinically relevant information was revealed according to the learner's line of questioning rather than provided in a single response. Scenario content was mapped to the UKM Year 3 Internal Medicine learning outcomes and competencies commonly assessed in Objective Structured Clinical Examinations and workplace-based assessments (27). Communication features included colloquial language, incomplete disclosure, variable health literacy, uncertainty, and contextually appropriate emotional expression. Such features are consistent with established design considerations for authentic virtual patients (12). In this study, they were specified in the scenario templates by clinical educators and subsequently evaluated by subspecialty SMEs.

The research team selected five evaluation domains to reflect the clinical content and conversational quality expected in UKM Year 3 history-taking (27), consistent with published virtual-patient and health-care LLM evaluation literature (13, 29–31). Clinical relevance assessed whether the inquiry or response was appropriate to the presenting problem and stage of the consultation. Clinical accuracy assessed whether the medical information was correct and internally consistent with the assigned diagnosis, symptoms, patient profile, and preceding dialogue. Completeness assessed whether sufficient relevant information was elicited or provided without important omissions or inappropriate over-disclosure. Clarity and coherence assessed comprehensibility, logical organisation, and continuity with the conversational context. Human-likeness assessed whether the language, phrasing, uncertainty, and pattern of information disclosure resembled a plausible clinician–patient interaction.

The operational definitions of the history-taking domains are presented in Table 1.

**Table 1:** History-taking domains.

Component	Operational description
Presenting complaint	Primary symptom or concern prompting the consultation
History of presenting illness	Structured exploration of onset, duration, progression, severity, associated symptoms, aggravating or relieving factors, and effects on daily functioning
Past medical history	Previous illnesses, chronic conditions, admissions, and relevant investigations
Past surgical history	Previous procedures and associated complications
Medication history	Current and recent medicines, dose, adherence, adverse effects, and complementary treatments where relevant
Allergy history	Drug, food, or environmental allergies and associated reactions
Family history	Relevant hereditary conditions or similar illnesses in immediate family members
Social history	Occupation, smoking, alcohol use, living arrangements, travel, functional status, and other relevant contextual factors
Review of systems	Systematic screening for additional symptoms across organ systems

The history-taking domains and operational descriptions were developed specifically for this study and aligned with the Universiti Kebangsaan Malaysia Year 3 Internal Medicine curriculum and standard clinical history-taking guidance (27, 28). They were not reproduced verbatim from a previously published framework.

Figure 2 illustrates how the principal history-taking domains were integrated into the structured simulation framework.

**Figure 2:**
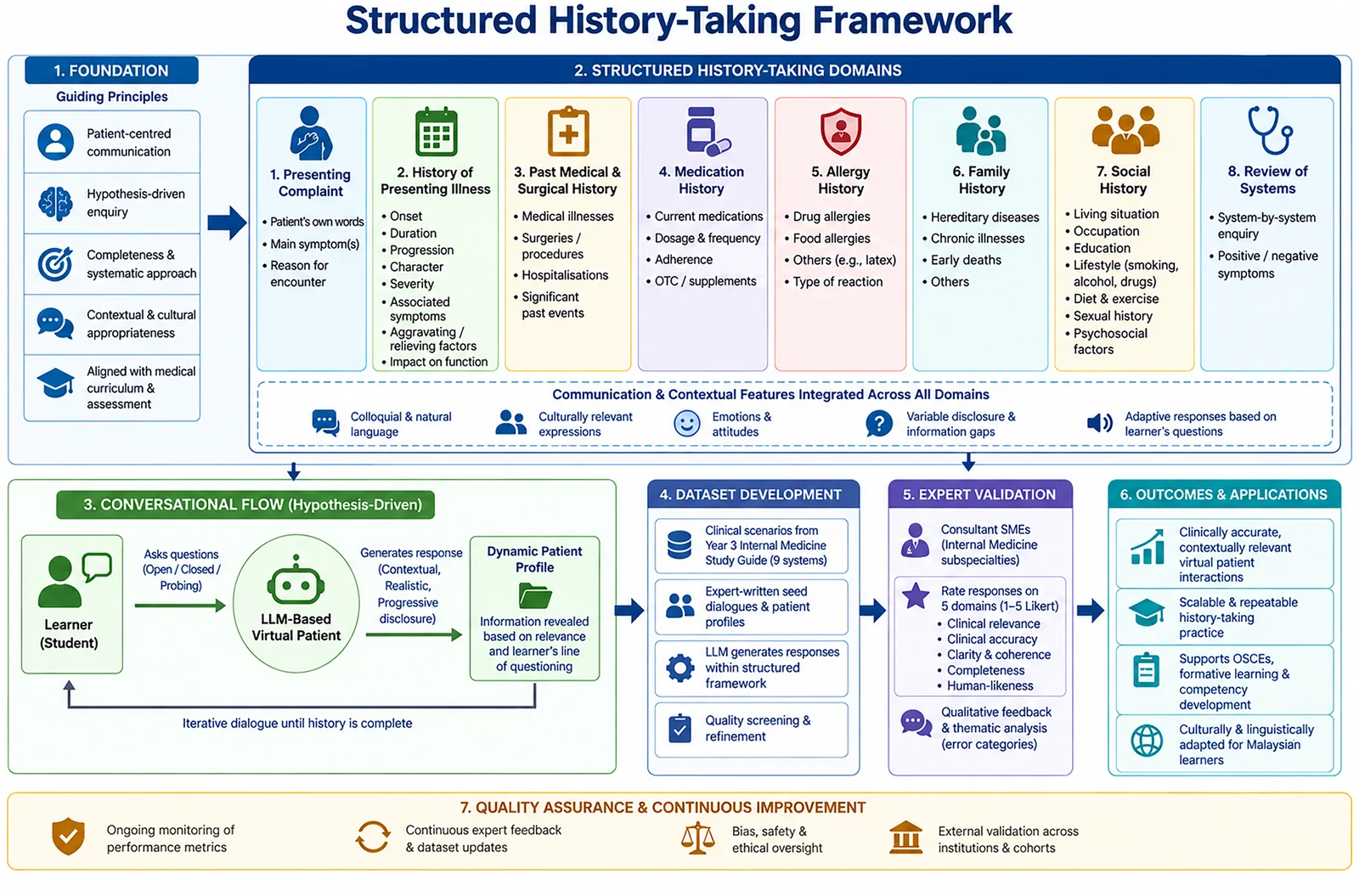
Structured history-taking framework.

#### Artificial intelligence response generation

A standardised prompt framework was developed to generate simulated clinical dialogue across a broad range of scenarios. Each prompt incorporated the assigned diagnosis, history-taking intent, patient demographic and clinical profile, health-literacy level, emotional disposition, social context, and information available at that stage of the encounter.

Simulated clinical dialogue was generated using GPT-5 (OpenAI), accessed through the ChatGPT web interface on 7 August 2025. Prompts followed a standardised template developed by the clinical author team and incorporated the assigned diagnosis, history-taking intent, patient profile, health-literacy level, emotional disposition, social context, and information available at the relevant stage of the clinical encounter. The complete generation prompt template and scenario-parameter structure are provided in Supplementary Methods S1.

Because the responses were generated through the ChatGPT web interface, model-level decoding parameters such as temperature, top-p, top-k, and random seed were not configurable or available to the authors. This limitation has now been stated explicitly to support transparent reporting and interpretation of the dataset-generation process.

The LLM was instructed to generate a simulated clinician inquiry and a corresponding response from the perspective of the assigned patient. Progressive disclosure was operationalised by limiting each patient response to information contained in the patient profile and relevant to the question asked. The simulated patient was not expected to disclose all clinically relevant information in a single response.

When a clinician inquiry requested information that was not contained in the patient profile, the model was instructed not to invent additional clinical facts. Instead, it was prompted to maintain the interaction by asking the learner to clarify the purpose or relevance of the question, for example, “Why do you ask?” This approach was intended to resemble facilitated problem-based learning, in which information outside the predefined case package is not supplied as an unsupported fact. Clinical specialists defined the scenario content and its informational boundaries. Ambiguous or uncertain elements were discussed with other members of the research team and resolved through specialist clinical input.

Unlike a static script, the prompt design allowed information to be revealed incrementally as the simulated consultation progressed. Scenario templates varied the detail of symptom descriptions, health-literacy level, use of colloquial expressions, emotional responses, degree of uncertainty, and amount of information disclosed during each conversational turn. These specifications were developed by clinical educators on the author team and subsequently assessed by subspecialty SMEs during their review of the generated exchanges.

The clinical scenarios were derived from the UKM Year 3 Internal Medicine learning outcomes and common presentations encountered in Malaysian clinical practice (27). The scenarios covered nine clinical systems: cardiovascular, respiratory, neurological, endocrine, nephrology, gastrointestinal, haematology, rheumatology, and infectious diseases.

English and Malay versions were generated to support bilingual use, with colloquial and locally meaningful expressions incorporated where appropriate. Bilingual capability and code-switching were treated as related but distinct concepts. Bilingual capability referred to the generation of dialogue in both English and Malay, including separate or parallel versions of the same clinical exchange. Code-switching referred to alternation between English and Malay within an individual utterance or across conversational turns. Code-switched exchanges were not separately labelled or quantitatively evaluated. Claims regarding language adaptation were therefore limited to bilingual English–Malay capability and sociolinguistic localisation rather than demonstrated code-switching performance.

Supplementary Table S1 presents an original Malay chatbot-generated interaction alongside an English translation prepared to aid readers' understanding. It therefore does not demonstrate code-switching within a single utterance or conversational turn.

Through iterative prompting and scenario refinement, 63,250 AI-generated dialogue entries were produced. Each entry comprised a simulated doctor inquiry and its corresponding patient response and was reviewed for formatting consistency before inclusion in the dataset repository.

#### Dataset structure

The dataset was organised in a structured tabular format. One dialogue entry represented one simulated clinician inquiry and its corresponding AI-generated patient response. This paired exchange constituted the unit of descriptive analysis. Multiple dialogue entries could originate from the same simulated consultation and were linked through a session identifier.

The dataset fields included the session identifier, medical topic, clinical diagnosis, history-taking intent, patient profile, clinician inquiry, AI-generated patient response, and relevant conversational metadata. The number of questions within each simulated consultation was not fixed because consultation length depended on the clinical scenario, patient profile, history-taking intent, and sequence of inquiries.

The simulated consultations did not end at a predefined diagnostic-decision threshold. Diagnostic decision-making and the number of questions required for a clinician to reach a diagnosis were not evaluated as outcomes in this model-development study.

The dataset variables and their operational definitions are presented in Table 2.

**Table 2:** Dataset structure and variable definitions.

Variable	Operational definition
Session ID	Identifier linking exchange rows belonging to the same simulated consultation
Medical topic	Curriculum-aligned internal medicine topic or clinical system
Clinical diagnosis	Assigned diagnosis, presenting condition, or clinical context
Intent	History-taking component targeted by the exchange, such as presenting complaint, history of presenting illness, medication history, social history, or review of systems
Role or patient profile	Simulated demographic and clinical background, health-literacy level, emotional disposition, social context and symptom profile
Doctor input	One simulated clinician question associated with the assigned history-taking intent and stage of the consultation
Patient response	A context-dependent simulated patient reply generated from the assigned patient profile, clinical scenario, preceding conversational context, and information available at that stage of the interaction. Depending on the scenario and inquiry, the response could include symptom details, uncertainty, selective disclosure, lay terminology, and locally relevant English or Malay expressions.

#### Subject matter expert validation

The evaluation panel comprised consultant-level SMEs from cardiology, respiratory medicine, neurology, endocrinology, nephrology, gastroenterology, haematology, rheumatology, and infectious diseases. All SMEs were clinically active and involved in undergraduate or postgraduate medical teaching. The validation roster assigned one SME to cardiovascular medicine, four to respiratory medicine, one to neurology, three to endocrinology, one to nephrology, four to gastroenterology, two to haematology, five to rheumatology, and three to infectious diseases. Experts reviewed entries within their respective subspecialty areas.

The validation process was conducted across three specialty-focused workshop cycles initiated on 23 July 2025, 8 September 2025, and 23 September 2025. SMEs were given until 30 October 2025 to complete the assigned assessments. The master validation record retained the number of validated entries at the clinical-system level but did not retain a complete per-individual workload record for each SME; consequently, the exact number of entries personally reviewed by each individual evaluator could not be reconstructed.

Validation was conducted using specialty-specific review and consensus processes. Generated doctor inquiries and patient responses were assessed against their assigned clinical scenario and history-taking context. Where more than one SME contributed within a clinical system, disagreements were discussed verbally until consensus was reached. Each dialogue entry therefore retained one final holistic score for the doctor inquiry and one for the corresponding patient response. Independent pre-consensus ratings, individual disagreement-level data, and complete reviewer-level rating records were not retained; consequently, inter-rater reliability could not be calculated retrospectively.

Of the 63,250 generated dialogue entries, 59,184 (93.6%) underwent SME evaluation. The remaining 4,066 entries were not advanced to validation because they were incomplete, duplicated, invalid, technically corrupted, or lacked sufficient contextual information.

#### Evaluation criteria

The Phase 1 history-taking framework and dialogue content were generated specifically for this study using an LLM under clinical educator guidance and were not adopted from a textbook or previous study. The five evaluation domains were selected by the research team because they reflected the expected clinical content and conversational quality of undergraduate history-taking interactions within the existing UKM Year 3 Internal Medicine curriculum (27). The criteria were also reviewed for consistency with standard clinical history-taking guidance and published approaches to designing and evaluating virtual patients and LLM-based clinical simulations (12, 13, 28–31).

Clinical relevance, clinical accuracy, clarity and coherence, completeness, and human-likeness informed a single author-developed holistic score from 1 to 5 rather than five independent subscale scores. Clinical relevance and accuracy addressed whether an exchange was clinically appropriate and consistent with the assigned scenario. Clarity and coherence addressed its comprehensibility and logical flow. Completeness addressed whether sufficient relevant information was elicited or provided without inappropriate over-disclosure. Human-likeness addressed whether the language, phrasing, degree of uncertainty, and pattern of information disclosure resembled a plausible clinician–patient interaction.

The same holistic rubric was applied separately to the simulated doctor inquiry and corresponding patient response. Criterion-level scores were not recorded. SMEs received standardised operational anchors for each score level and could provide free-text comments identifying clinical, linguistic, contextual, or conversational limitations. These comments supplemented the holistic scores and were not treated as a separate psychometric instrument.

The operational anchors are presented in Table 3.

**Table 3:** Holistic SME scoring rubric.

Score	Descriptor	Operational anchor
1	Very poor	Major clinical or contextual errors; irrelevant, contradictory, nonsensical, or unusable for history-taking practice
2	Poor	Partly relevant but contains important inaccuracies, omissions, ambiguity, or unrealistic language that substantially reduces educational value
3	Acceptable	Generally relevant and clinically plausible but generic, incomplete, awkwardly phrased, or insufficiently realistic
4	Good	Clinically appropriate, coherent, and sufficiently complete, with only minor omissions or linguistic limitations
5	Excellent	Clinically accurate, context-specific, clear, appropriately complete, and convincingly natural and authentic for the assigned conversational role

Clinical relevance, accuracy, clarity and coherence, completeness, and human-likeness informed one holistic score rather than five independent criterion-level scores.

#### Ethical considerations

This study involved the development and validation of AI-generated clinical dialogue for educational and model-development purposes. No real patient data, identifiable patient information, or student-performance data were used. The simulated clinical scenarios were generated from curriculum-aligned internal medicine learning outcomes and did not represent actual patient encounters.

SMEs evaluated AI-generated dialogue within their respective subspecialty areas. All participating SMEs were informed of the study objectives and the nature of their involvement before participation and provided informed consent. No compensation was provided. Expert feedback was analysed and reported in aggregate, and individual evaluators were not identified. Study data were stored securely and used only for research and model-development purposes.

Ethical approval was obtained from the Universiti Kebangsaan Malaysia Ethics Review Committee under approval number JEP-2025-333.

### Phase 2: dataset preparation for fine-tuning

#### Data cleaning

Following SME validation, the dataset underwent systematic clinical-quality and mechanical screening before model development. Holistic scores of 1 or 2 were considered below the predefined acceptability threshold, whereas scores of 3–5 were considered eligible for inclusion. Because each dialogue entry comprised a paired simulated doctor inquiry and patient response, the entire paired entry was excluded when either component received a holistic score of 1 or 2.

Mechanical filtering subsequently removed incomplete records, duplicate entries, invalid responses, technically corrupted outputs, and entries without sufficient contextual information. Eligibility was determined using the SME scores rather than by retrospectively reclassifying the free-text comments. Factual or clinically inappropriate entries were therefore excluded when either associated holistic score fell below the predefined threshold.

After clinical-quality and mechanical filtering, 58,886 of the 59,184 SME-evaluated dialogue entries were retained, corresponding to 298 post-validation exclusions. The available processing record retained the aggregate number of exclusions but did not permit reliable retrospective separation of this total into individual clinical-quality and mechanical exclusion categories. The score distribution of the retained dataset is reported in Supplementary Table S2.

The final model-development dataset therefore contained 58,886 dialogue entries associated with 2,497 simulated patient profiles across the nine clinical systems.

#### Data structuring

The cleaned dataset was reformatted into ShareGPT multi-turn dialogue format for supervised fine-tuning. This format represented sequential exchanges between clinician and patient roles and allowed entries from the same simulated consultation to remain contextually linked.

Each training instance included the dialogue together with its clinical system, diagnosis, patient profile, medical topic, and relevant contextual information. This structure was intended to help the model associate clinical and demographic contexts with appropriate conversational patterns, progressive information disclosure, uncertainty management, and language use.

### Phase 3: model development

#### Base model

The base checkpoint was mesolitica/Malaysian-Llama-3.1-8B-Instruct-v0.1, obtained from Hugging Face. This 8.07-billion-parameter checkpoint is a Malaysian-adapted derivative of Meta Llama 3.1 8B Instruct and was used under the Llama 3.1 Community License. It was selected because of its support for English–Malay conversational tasks and its computational feasibility for academic model development.

#### Parameter-efficient fine-tuning

Low-Rank Adaptation (LoRA) was used for parameter-efficient fine-tuning (32) within a 4-bit QLoRA workflow. The LoRA configuration used a rank of 16, alpha of 16, dropout of 0, no bias, and the q_proj, k_proj, v_proj, o_proj, gate_proj, up_proj, and down_proj target modules, resulting in 41,943,040 trainable parameters (0.52%). Training used the adamw_8bit optimiser, a learning rate of 2 × 10^−4^, a linear schedule, five warm-up steps, one epoch, an effective batch size of 32 derived from a per-device batch size of 8 and four gradient-accumulation steps, and a maximum sequence length of 2,048 tokens. Loss was applied only to assistant or simulated-patient turns, while clinician turns were masked.

The 2,497 simulated patient profiles were partitioned through stratified random sampling across the nine clinical systems using a fixed data-split seed of 42. The resulting partitions comprised 1,997 training profiles, 250 validation profiles, and 250 held-out test profiles. All dialogue entries associated with the same patient profile and simulated consultation were assigned wholly to one partition.

Fine-tuning was conducted with a training seed of 3,407 on one NVIDIA Tesla T4 GPU with 14.7 GB of memory. The implementation used Unsloth 2025.11.6, Transformers 4.56.2, TRL 0.22.2, PyTorch 2.9.1 + cu128, and Python 3.11.13. Validation was performed every 30 optimisation steps. Early stopping was not applied; instead, the checkpoint with the lowest observed validation loss was restored after training. The training run comprised 63 optimiser steps and required approximately 2 h and 14 min.

The 250 held-out profiles and all associated dialogue content were excluded from both model fitting and checkpoint selection. The 250 validation profiles were used only for validation-loss monitoring and checkpoint selection and were not included in the held-out benchmark.

The complete training configuration, software environment, validation losses, and LoRA adapter parameters are provided in Supplementary Table S3. Additional technical materials relating to the fine-tuning procedure are available from the corresponding author upon reasonable request, subject to institutional governance and applicable licensing requirements.

### Phase 4: model benchmarking and evaluation

#### Internal holdout benchmark Set

The same 250 held-out patient profiles were used for internal descriptive benchmarking across all model configurations. Because all dialogue content associated with these profiles had been excluded from fine-tuning, the benchmark avoided direct patient-profile-level overlap between the fine-tuning and holdout partitions. However, the held-out profiles originated from the same overall corpus-development process and therefore did not constitute an independently clinician-authored external test set.

The purpose of this phase was to compare model configurations under a common set of internal test inputs to support model selection and technical development. It was not designed to demonstrate external generalisability, clinical superiority, or educational effectiveness.

The four configurations comprised the Malaysian-adapted Llama 3.1 8B Instruct base model with zero-shot prompting, the base model with 5-shot prompting, the base model with 15-shot prompting, and the LoRA fine-tuned model with zero-shot prompting. For each held-out profile, the configuration received the virtual-patient system prompt and the full preceding consultation and generated the final simulated-patient utterance. Generation was capped at 50 tokens because the reference responses were typically approximately 10-19 words; all other generation settings used each model's packaged generation configuration. The same 250 profile-linked test inputs were presented to each configuration, resulting in 1,000 generated patient responses.

#### Automated scoring pipeline

An LLM-as-a-judge approach was used for internal descriptive benchmarking (33, 34). Google Gemini 2.5 Flash was accessed through OpenRouter using the identifier google/gemini-2.5-flash and applied a holistic 5-point patient-response rubric aligned with the SME evaluation framework.

The evaluator was few-shot prompted using 120 SME-scored exemplars, comprising 24 exemplars for each holistic score level from 1 to 5. All exemplars were selected exclusively from the training partition; none originated from the validation partition or the 250 held-out test profiles. The exemplars were used to standardise the evaluator's interpretation of clinical relevance, clinical accuracy, clarity and coherence, completeness, and human-likeness. The judge was instructed to return only one holistic patient-response score without accompanying reasoning.

Temperature, top-p, top-k, and maximum-output-token values were not explicitly set in the OpenRouter judge request and were therefore passed through to the upstream provider's defaults. Reasoning was enabled with a requested budget_tokens value of 10,000. The exact access date was not retained in the available technical log. These limitations are reported explicitly rather than assigning retrospectively inferred parameter values.

Supplementary Methods S2 describes the benchmarking and automated-scoring pipeline. Additional materials relating to the evaluator prompt, scoring anchors, per-item input structure, and few-shot exemplars are available from the corresponding author upon reasonable request, subject to institutional governance and applicable licensing requirements.

Automated evaluation was intended to provide a consistent internal benchmark for comparing model configurations. It was not treated as equivalent to blinded human assessment or clinical validation of post-fine-tuning outputs. No clinician ratings of the 1,000 benchmark responses were collected in the present study, and human–machine agreement could therefore not be calculated. The benchmarking analysis did not include uncertainty estimates or inferential hypothesis testing.

#### Statistical and qualitative analysis

The unit of quantitative analysis for SME evaluation was one dialogue entry comprising a simulated doctor inquiry and its corresponding patient response. A total of 59,184 dialogue entries underwent SME validation. Doctor-inquiry and patient-response scores were analysed separately.

Holistic scores were summarised by clinical system and overall using the number of validated entries, mean, standard deviation, median, and interquartile range. The interquartile range was expressed as the 25th to 75th percentile. Means and standard deviations were retained to facilitate comparison with the original analysis, while medians and interquartile ranges were reported because the scores were bounded ordinal ratings with potentially skewed distributions. Missing values were not imputed.

Because the number of entries differed across clinical systems, an item-weighted mean and system-equal mean were calculated. The item-weighted mean included all valid ratings, allowing systems with more entries to contribute proportionally more. The system-equal mean was calculated by averaging the nine system-specific means, thereby assigning equal weight to each clinical system. The two estimates were compared descriptively to assess the potential influence of unequal system sizes. System-specific findings were interpreted descriptively because different subspecialty panels evaluated their respective clinical systems.

No inferential statistical tests were used to compare clinical systems or benchmark configurations. Multiple entries could originate from the same simulated consultation, and the available data did not support modelling of within-session clustering or reviewer effects. Treating each dialogue entry as an independent observation for inferential testing could therefore have produced artificially precise estimates. Differences between clinical systems and model configurations were interpreted descriptively.

Free-text SME comments were reviewed iteratively and coded inductively to identify recurring clinical, linguistic, contextual, and conversational limitations. Related codes were grouped into broader themes and analysed separately for simulated doctor inquiries and patient responses. Representative Malay comments were retained and accompanied by semantic English translations reviewed by the bilingual author team. The qualitative analysis was intended to characterise recurrent error patterns rather than estimate their prevalence.

## Results

### Dataset generation and expert validation

A total of 63,250 paired clinical dialogue entries were generated across the nine internal medicine systems. Each entry comprised a simulated doctor inquiry and its corresponding patient response. Of these, 59,184 entries (93.6%) underwent consultant-level SME evaluation. Following application of the clinical-quality threshold and mechanical cleaning criteria, 298 post-validation entries were excluded and 58,886 entries associated with 2,497 simulated patient profiles were retained for model development. The retained corpus contained only entries whose paired doctor-inquiry and patient-response scores met the eligibility rule described in the Methods; the retained-score distribution is reported in Supplementary Table S2.

Dataset partitioning was performed at the simulated patient-profile level. Of the 2,497 profiles, 1,997 were allocated to the training partition, 250 to the validation partition, and 250 to the held-out test partition. All dialogue entries associated with the same patient profile remained within one partition. The validation profiles supported checkpoint selection, whereas the held-out profiles were used only for the internal descriptive benchmark.

### Quantitative SME validation

Table 4 presents the descriptive SME scores across the nine clinical systems. The number of evaluated entries differed substantially between systems, ranging from 2,843 cardiovascular entries to 10,979 infectious disease entries. The gastrointestinal system contributed 10,211 entries, compared with 5,561 entries from nephrology. Consequently, systems containing more entries contributed proportionally more to the item-weighted overall scores.

**Table 4:** SME scores by clinical system.

Clinical system	Evaluated entries	Doctor inquiry, mean (SD)	Doctor inquiry, median (Q1–Q3)	Patient response, mean (SD)	Patient response, median (Q1–Q3)
Haematology	3,655	4.94 (0.27)	5.00 (5.00–5.00)	4.94 (0.31)	5.00 (5.00–5.00)
Rheumatology	5,510	4.88 (0.50)	5.00 (5.00–5.00)	4.86 (0.52)	5.00 (5.00–5.00)
Neurology	5,591	4.82 (0.74)	5.00 (5.00–5.00)	4.85 (0.70)	5.00 (5.00–5.00)
Gastrointestinal	10,211	4.87 (0.47)	5.00 (5.00–5.00)	4.89 (0.44)	5.00 (5.00–5.00)
Nephrology	5,561	4.72 (0.62)	5.00 (5.00–5.00)	4.89 (0.48)	5.00 (5.00–5.00)
Endocrinology	10,046	4.43 (0.62)	4.00 (4.00–5.00)	4.41 (0.65)	4.00 (4.00–5.00)
Respiratory	4,788	4.15 (1.20)	4.00 (4.00–5.00)	4.17 (1.20)	5.00 (4.00–5.00)
Infectious disease	10,979	4.06 (1.07)	4.00 (4.00–5.00)	4.29 (0.87)	4.00 (4.00–5.00)
Cardiovascular	2,843	3.99 (0.10)	4.00 (4.00–4.00)	3.99 (0.14)	4.00 (4.00–4.00)
Total	59,184				
Mean (SD)		4.52 (0.82)	5.00 (4.00–5.00)	4.58 (0.76)	5.00 (4.00–5.00)
System-equal mean		4.54		4.59	

Clinical-system counts represent entries that underwent SME validation and sum to 59,184. Item-weighted means were calculated across all valid holistic ratings, whereas system-equal means were calculated by assigning equal weight to each of the nine clinical systems. Missing ratings were not imputed. Cross-system comparisons were descriptive because different subspecialty panels evaluated their respective systems. Scores ranged from 1 to 5, with higher scores indicating better overall quality. Medians are presented with Q1-Q3. SD, standard deviation; SME, subject matter expert.

Across all valid ratings, the item-weighted mean score was 4.52 (SD: 0.82) for simulated doctor inquiries and 4.58 (SD: 0.76) for simulated patient responses. The corresponding medians were 5.00 (Q1–Q3: 4.00–5.00) for both dialogue roles. When each clinical system was assigned equal weight, the mean scores were 4.54 and 4.59, respectively. The item-weighted and system-equal means differed by 0.02 for doctor inquiries and 0.01 for patient responses, suggesting that the overall estimates were not substantially altered when equal weight was assigned to each clinical system.

Mean scores exceeded 4.40 for both dialogue roles in haematology, rheumatology, neurology, gastrointestinal medicine, nephrology, and endocrinology. Haematology had the highest observed mean scores, at 4.94 for both simulated doctor inquiries and patient responses. Gastrointestinal, neurological, rheumatological, nephrological, and haematological entries had median scores of 5.00 for both dialogue roles. Cardiovascular entries had the lowest observed mean scores, at 3.99 for both simulated doctor inquiries and patient responses. These differences were interpreted descriptively because scenario complexity, case composition, symptom diversity, reviewer expectations, and scoring severity could not be separated from differences between the subspecialty panels. The system-specific findings were therefore used to identify areas requiring targeted refinement rather than to rank the clinical systems.

### Qualitative SME feedback

Free-text SME comments identified recurring limitations in the AI-generated clinical dialogue. Comments relating to simulated doctor inquiries and patient responses were analysed separately because the two conversational roles demonstrated different patterns of limitations. All representative comments originally provided in Malay are accompanied by semantic English translations.

### Simulated doctor inquiries

Four broad themes were identified in the simulated doctor inquiries: flawed questioning technique, incomplete clinical probing, irrelevance and redundancy, and linguistic and terminology errors.

Flawed questioning techniques included compound questions, premature use of closed questions, and poor sequencing. Some inquiries combined several symptoms or clinical elements within a single question, making them difficult for a simulated patient to answer naturally. SMEs also indicated that broad, open-ended questions should generally precede focused or review-of-systems questions.

Incomplete clinical probing involved the omission of relevant history-taking components, including surgical, medication, occupational, travel, and functional histories. SMEs also identified clinically relevant questions that appeared too late in the consultation.

Irrelevance and redundancy included repeated questions and inquiries unrelated to the presenting complaint or relevant differential diagnoses. Linguistic and terminology errors included incomplete sentence structures, grammatical problems, and medical terminology considered unsuitable for communication with a layperson. The themes and representative SME comments are presented in Table 5.

**Table 5:** Themes identified in simulated doctor inquiries .

Theme	Subtheme	Representative SME comments
Flawed questioning technique	Compound questioning	“Too many symptoms in one question”; “Banyak sangat gejala dalam satu soalan, susah pesakit nak jawab” [English: “There are too many symptoms in one question, making it difficult for the patient to answer”]; “Soalan gabungan” [English: “Combined questions”]
Flawed questioning technique	Premature closed questions	“Ask general first”; “Untuk systemic review, better to ask a general open-ended question, iaitu ‘Ada masalah atau gejala lain yang encik nak beritahu?’” [English: “For the review of systems, it would be better to begin with a general open-ended question, such as ‘Are there any other problems or symptoms that you would like to tell me about?’”]
Flawed questioning technique	Poor sequencing	“Questions should come after the joint-pain questions”; “This should be asked earlier as part of the associated symptoms in the history of presenting illness”
Incomplete clinical probing	Missing clinical-history components	“Perlu tanya sejarah pembedahan” [English: “The surgical history should be asked”]; “Batuk berkahak tak?” [English: “Is the cough productive?”]; “Bagaimana kawalan gula?” [English: “How well controlled is the blood glucose?”]
Incomplete clinical probing	Missing social or functional history	“Ada melancong ke mana-mana?” [English: “Have you travelled anywhere?”]; “Encik kerja apa?” [English: “What is your occupation?”]; “Adakah sakit mengganggu aktiviti harian encik?” [English: “Does the pain interfere with your daily activities?”]
Irrelevance and redundancy	Repeated questions	“Soalan berulang” [English: “Repeated question”]; “Berulang” [English: “Repetitive”]; “Already asked”
Irrelevance and redundancy	Clinically irrelevant questions	“Tak relevan” [English: “Not relevant”]; “Remove”; “Void”
Linguistic and terminology errors	Inappropriate jargon	“Penyakit kelamin ganti STI” [English: “Use the lay term ‘penyakit kelamin’ instead of the abbreviation STI”]
Linguistic and terminology errors	Grammatical or structural problems	“Ayat tergantung” [English: “The sentence is incomplete”]; “Salah bahasa” [English: “Incorrect language use”]; “Ayat tak lengkap” [English: “Incomplete sentence”]

### Simulated patient responses

Four broad themes were identified in the simulated patient responses: unrealistic patient persona, contextual and terminological inaccuracy, factual errors, and technical generation failures.

Over-explanation was a recurring concern, with some simulated patients providing unusually detailed or clinically complete answers rather than disclosing information progressively. SMEs also identified unnatural symptom descriptions, including anatomical or directional descriptions that did not reflect how patients would ordinarily describe the location or radiation of pain. Some responses did not directly or completely answer the clinician's question.

Contextual and terminological limitations included insufficient use of locally familiar expressions and excessive use of formal or medical terminology. SMEs recommended more familiar expressions such as “ubat kencing manis” for diabetes medicine, “seram sejuk” for chills, and “mengantuk” for drowsiness, depending on the clinical context. Factual errors included incorrect medication terminology, while technical generation failures included incomplete or truncated responses. The themes and representative comments are presented in Table 6.

**Table 6:** Themes identified in simulated patient responses.

Theme	Subtheme	Representative SME comments
Unrealistic patient persona	Over-explanation	“The patient would not usually describe all symptoms in one sentence”; “Pesakit selalu jawab satu gejala sahaja” [English: “Patients usually describe only one symptom at a time”]
Unrealistic patient persona	Unnatural symptom descriptions	“Selalunya pesakit jawab di mana sakit dan merebak ke bahagian mana, bukan arah” [English: “Patients usually describe where the pain is and where it spreads, rather than using anatomical directions”]
Unrealistic patient persona	Failure to answer the question	“Tidak menjawab soalan” [English: “The question was not answered”]; “Pesakit tak jawab semua soalan” [English: “The patient did not answer all parts of the question”]
Contextual and terminological inaccuracy	Limited local colloquial language	“Change to ‘ubat kencing manis’” [English: “Use the common patient term ‘diabetes medicine’”]; “Usually they say ’seram sejuk’” [English: “Patients commonly describe chills as ‘seram sejuk’”]; “‘Lalok’ = ‘mengantuk’?” [English: “Should ‘lalok’ be replaced with the clearer term ‘drowsy’?”]
Contextual and terminological inaccuracy	Inappropriate medical jargon	“‘Flare’ = ‘aktif’” [English: “Replace ‘flare’ with the more understandable Malay term ‘active’”]; “‘Teropong usus’” [English: “An intestinal scope or endoscopic examination”]; “‘Kurang darah’” [English: “Low blood or anaemia”]; “‘Nampak pucat’” [English: “Looks pale”]
Factual errors	Incorrect medication terminology	“There is no medication named ‘uric acid’”
Technical generation failure	Truncated responses	“Tak lengkap” [English: “Incomplete”]

### Model benchmarking

The same internal holdout set of 250 simulated patient profiles was used to descriptively benchmark the four model configurations. The LoRA fine-tuned model achieved the highest observed mean patient-response score, at 4.772/5. The zero-shot Malaysian-adapted base model achieved a mean score of 4.684/5, while the 5-shot and 15-shot prompting configurations achieved mean scores of 3.992/5 and 3.980/5, respectively (Table 7).

**Table 7:** Benchmark scores by model configuration.

Model configuration	Prompting strategy	Holdout patient profiles, n	Mean patient-response score
LoRA fine-tuned Llama 3.1 8B Instruct model	Zero-shot	250	4.772
Malaysian-adapted Llama 3.1 8B Instruct base model	Zero-shot	250	4.684
Malaysian-adapted Llama 3.1 8B Instruct base model	5-shot prompting	250	3.992
Malaysian-adapted Llama 3.1 8B Instruct base model	15-shot prompting	250	3.980

The holdout set comprised approximately 10% of the 2,497 simulated patient profiles. The same 250 profiles were evaluated across all four configurations, and all dialogue content associated with these profiles was excluded from fine-tuning. The profiles nevertheless originated from the same overall corpus-development process and therefore constituted an internal rather than external test set. Patient responses were evaluated using Gemini 2.5 Flash as an automated LLM judge guided by the holistic 5-point rubric. No blinded human evaluation of these post-fine-tuning benchmark outputs was performed. Higher scores indicate better performance within this internal automated benchmark only; the comparisons do not establish statistical, clinical, educational, or external superiority between configurations.

These values describe the observed ordering of the four configurations within an internal automated benchmark. Although patient-profile-level partitioning prevented direct inclusion of the held-out profiles in fine-tuning, the holdout cases originated from the same overall corpus-development process and were not independently authored external clinical cases. In addition, scoring was performed using an automated LLM judge without blinded clinician adjudication. The observed numerical differences therefore should not be interpreted as evidence of statistical, clinical, educational, or externally generalisable superiority of the fine-tuned model.

## Discussion

### Principal findings

This study demonstrates the feasibility of developing a clinically reviewed and culturally and linguistically adapted English–Malay LLM framework for simulated clinical history-taking. The generally high holistic SME scores suggest that many of the generated doctor inquiries and patient responses were considered clinically and conversationally appropriate. However, these scores should be interpreted alongside the qualitative findings. Expert comments identified clinically and educationally important limitations involving flawed questioning techniques, incomplete clinical probing, irrelevance and redundancy, linguistic and terminological inaccuracies, unrealistic patient behaviour, factual errors, and technical generation failures. Thus, favourable overall scores did not eliminate the need for detailed qualitative clinical review.

The item-weighted mean scores were 4.52/5 for simulated doctor inquiries and 4.58/5 for patient responses, while the corresponding system-equal means were 4.54 and 4.59. The small numerical differences between the item-weighted and system-equal means suggest that assigning equal weight to each clinical system did not substantially alter the overall descriptive estimates. Nevertheless, the lower observed scores in the cardiovascular, respiratory, and infectious disease systems should not be interpreted as definitive evidence of poorer model performance in those specialties. Differences in scenario complexity, case composition, symptom diversity, reviewer expectations, and scoring severity among the subspecialty panels could not be separated analytically. The system-specific results are therefore more appropriately used to identify areas requiring targeted refinement than to rank model performance across clinical systems.

The LoRA fine-tuned model achieved the highest observed mean score in the internal automated benchmark. This finding may reflect closer alignment between the fine-tuned model and the clinical, linguistic, and conversational patterns represented in the development corpus. However, the benchmark was intended for internal model comparison and selection rather than external or clinical validation. Although the held-out patient profiles were excluded from fine-tuning, they originated from the same overall corpus-development process. Furthermore, the benchmark relied on a single automated LLM judge and did not include confidence intervals, inferential testing, or blinded clinician assessment of the post-fine-tuning outputs. The observed results therefore do not establish the statistical, clinical, educational, or externally generalisable superiority of the fine-tuned model.

### Comparison with prior work

The findings contribute to the growing literature on LLM-based virtual patients and AI-supported clinical-skills education. Earlier studies have demonstrated strong LLM performance in medical knowledge assessments and clinical question-answering tasks while also identifying hallucinations, contextual inconsistency, variable reliability, and limited educational validation (15–20). Performance in knowledge-based tasks does not necessarily establish suitability for simulated patient interaction, where information disclosure, conversational continuity, linguistic authenticity, uncertainty, and emotional expression are also important.

More recent studies have examined purpose-built systems for clinical dialogue, history-taking practice, communication training, automated feedback, and structured model evaluation (30, 31, 35–37). These studies have emphasised evaluation consistency and human–AI agreement (30), scalable dialogue and feedback (31), user-centred design (35), standardised evaluation of virtual patients (36), and interactive clinical-skills training (37).

The present framework extends this work by combining a large curriculum-aligned dataset, consultant-level review across nine internal medicine systems, locally contextualised English–Malay content, qualitative analysis of recurrent limitations, parameter-efficient fine-tuning, and descriptive benchmarking within one development process. The findings reinforce the importance of combining quantitative model assessment with qualitative clinical review. A single numerical score may indicate overall acceptability but may not reveal specific educationally important problems, such as compound questioning, inappropriate sequencing, omitted clinical domains, excessive information disclosure, or unnatural patient language.

### Importance of cultural and linguistic adaptation

Effective clinical simulation requires more than literal translation. SME feedback showed that grammatically correct Malay responses could still sound excessively formal, contain unfamiliar medical terminology, or fail to resemble how patients commonly describe their symptoms. In Malaysian clinical practice, consultations may be conducted in English, Malay, or a combination of both languages and may incorporate colloquial and culturally familiar expressions (21, 22).

SME preferences for expressions such as “ubat kencing manis” for diabetes medicine and “seram sejuk” for chills illustrate the educational importance of sociolinguistic authenticity. These language choices may influence whether learners recognise a patient's intended meaning, ask appropriate clarification questions, respond to verbal cues, establish rapport, and translate everyday symptom descriptions into clinically meaningful information. A response can therefore be clinically accurate while remaining conversationally inappropriate for the intended patient profile.

Bilingual capability and code-switching should not be treated as interchangeable concepts. The present study evaluated English and Malay dialogue, including parallel-language content and locally adapted expressions. This supports bilingual response generation but does not establish the model's ability to produce or interpret natural code-switching. Code-switching involves alternation between English and Malay within the same utterance or across conversational turns, and such exchanges were not separately labelled or evaluated in this study.

General-purpose LLMs developed predominantly using high-resource language data may inadequately represent regional languages and culturally embedded patterns of clinical communication (21, 23–25). A model may consequently produce clinically plausible Malay dialogue while still requiring refinement in lay vocabulary, health-literacy level, indirect communication, culturally familiar symptom descriptions, and patterns of information disclosure. Furthermore, the present linguistic adaptation was evaluated primarily by clinicians. Evaluation involving patients, linguists, communication specialists, and medical learners would provide a broader assessment of sociolinguistic authenticity and comprehensibility.

### Fine-tuning and prompting strategies

The internal descriptive benchmark suggests that parameter-efficient fine-tuning may help align model outputs with the conversational patterns represented in the development corpus. The LoRA fine-tuned model achieved the highest observed mean automated score, whereas the 5-shot and 15-shot prompting configurations had lower observed means than the zero-shot base model. These results should not be interpreted as evidence that fine-tuning is clinically superior to prompt engineering or that the relative performance observed here will generalise to independently developed cases, other institutions, or student-facing use.

The observed differences may have been influenced by the selection, quality, ordering, and representativeness of the few-shot examples; prompt length; contextual interference; or alignment between the fine-tuning dataset and the automated evaluation criteria. The use of the same automated evaluator across all configurations may also have introduced evaluator-specific alignment bias. An automated judge may preferentially reward response characteristics that resemble its own interpretation of the rubric without necessarily reflecting the judgements of clinical educators, patients, or learners.

Prompt engineering remains useful for rapid prototyping and adaptation without model retraining. Fine-tuning, in contrast, may allow clinical patterns, patient profiles, progressive disclosure, and locally appropriate communication features to be incorporated more systematically. LoRA provides a computationally feasible approach to this form of targeted adaptation because it requires substantially fewer trainable parameters than full model fine-tuning (32). Future comparisons should use independently developed test scenarios, paired response-level analyses, uncertainty estimates, multiple evaluators, and blinded assessment of post-fine-tuning outputs by clinical educators and intended learners. Human–machine agreement should also be evaluated when both clinician and automated ratings are available for the same model outputs.

### Educational implications

The framework may provide opportunities for repeated, low-stakes history-taking practice before encounters with real patients. Bedside teaching, supervised clinical exposure, and standardised patient encounters remain essential, but digital simulation could supplement these approaches by allowing learners to practise structured enquiry, encounter different clinical presentations, and repeat consultations after reflection and feedback.

The error patterns identified by the SMEs could inform structured reflective learning. For example, a learner who asks, “Do you have fever, cough, breathlessness, chest pain, or wheezing?” could review the consultation transcript and recognise that several symptoms were combined within one question. The learner could then repeat the encounter by beginning with an open invitation and asking one focused question at a time. Similarly, learners who omit medication adherence, travel history, occupational exposure, or functional impact could compare their transcripts with an educator-approved, curriculum-aligned framework and identify specific areas for improvement.

A structured reflective process could involve completing a simulated consultation, reviewing the transcript, identifying compound, repeated, omitted, poorly sequenced, or inappropriately phrased questions, comparing the encounter with an educator-approved framework, formulating specific improvement goals, and repeating the scenario using a revised questioning approach. The original and repeated encounters could subsequently be compared using faculty feedback or an approved rubric.

These applications remain proposed educational uses rather than demonstrated outcomes. The present study did not involve learners and did not evaluate student confidence, history-taking completeness, communication performance, diagnostic reasoning, knowledge retention, OSCE performance, or transfer to real patient encounters. Although the dataset was aligned with the Year 3 Internal Medicine curriculum, the framework was not formally evaluated against validated OSCE station checklists or scoring standards. It should therefore not be assumed to improve OSCE preparedness or clinical competence without prospective learner-based evaluation.

### Implementation and scalability

The framework may help address some structural constraints affecting clinical-skills education. AI-supported simulation can provide repeatable clinical scenarios across learner cohorts while retaining a degree of conversational variability. Such systems may be particularly useful when access to standardised patients, simulation facilities, clinical placements, or specialist supervisors is limited. Parameter-efficient LoRA fine-tuning may also support technical scalability by reducing computational requirements compared with full model retraining (32).

Implementation should nevertheless remain educationally and clinically governed. The framework should function as a preparatory and supplementary learning resource rather than as a replacement for bedside teaching, faculty supervision, standardised patients, or authentic patient interaction. Faculty oversight remains necessary to ensure clinical accuracy, curriculum alignment, appropriate feedback, ethical use, accessibility, privacy, and responsible data governance.

The present study did not conduct a targeted clinician-adjudicated safety evaluation of post-fine-tuning model outputs and did not quantify the frequency of medication errors, diagnostic inaccuracies, fabricated clinical information, unsafe recommendations, or other clinically consequential hallucinations after fine-tuning. Likewise, refusal behaviour and model guardrails were not systematically stress-tested as formal study outcomes. Accordingly, the framework should not undergo unsupervised student-facing deployment on the basis of the present findings alone. Before implementation, a dedicated safety evaluation should include clinician review of sampled model outputs, explicit assessment of medication and diagnostic accuracy, testing of responses to out-of-scope or unsupported questions, and predefined procedures for educator monitoring, error reporting, content review, and periodic re-evaluation following model or software updates.

## Strengths and limitations

A principal strength of this study was the integration of curriculum-aligned dataset development, consultant-level SME review, qualitative error analysis, parameter-efficient fine-tuning, and descriptive model benchmarking. The inclusion of nine internal medicine systems and locally contextualised English–Malay content addresses an important gap in the development of non-English and culturally adapted virtual-patient frameworks. Partitioning at the patient-profile level also reduced the risk of direct leakage of dialogue content from the same simulated patient between the fine-tuning and holdout partitions.

Several limitations should be considered. The study was conducted at a single institution, and the scenarios, curricular priorities, communication expectations, and linguistic adaptations may reflect local teaching practices. The corpus was specifically developed for English–Malay clinical history-taking and should not be interpreted as representing the full multilingual diversity of Malaysian clinical encounters. Mandarin and other Chinese varieties, Tamil, and other languages used by Malaysian patients were not represented or evaluated in this study. External validity beyond Year 3 internal medicine education at UKM has therefore not been established.

Patient ethnicity was not systematically encoded as an analysable variable in the available dataset, and the study did not undertake a formal assessment of stereotyped representation or demographic bias within the simulated patient personas. It was therefore not possible to determine whether different Malaysian ethnic groups were proportionately or appropriately represented. Future datasets should prospectively define relevant demographic characteristics, include broader linguistic and sociocultural representation, and incorporate structured evaluation for stereotyped, biased, or otherwise inappropriate representations of patient groups.

The five evaluation criteria informed an author-developed holistic rubric rather than a previously validated psychometric instrument. Criterion-level scores were not recorded, and it was therefore not possible to analyse clinical relevance, clinical accuracy, clarity and coherence, completeness, and human-likeness as separate dimensions. The concentration of ratings toward the upper end of the scale may also have limited the rubric's ability to distinguish between high-quality entries.

Clinical-system sample sizes were unequal, and separate subspecialty panels evaluated their respective systems. Although the item-weighted and system-equal means were similar, observed system-level differences may still reflect differences in case composition, scenario difficulty, reviewer expectations, or scoring severity rather than differences in generated-dialogue quality alone.

Multiple dialogue entries could originate from the same simulated consultation, but within-consultation clustering was not modelled. Treating every entry as an independent observation for inferential testing could therefore have produced artificially precise estimates. For this reason, the quantitative findings were interpreted descriptively.

Entries were evaluated through specialty-specific review conducted across three workshop cycles initiated on 23 July 2025, 8 September 2025, and 23 September 2025, with SMEs given until 30 October 2025 to complete the assessments. The number of SMEs assigned varied across clinical systems. However, independent pre-consensus ratings, complete individual reviewer-level workload records, reviewer identifiers linked to every final rating, and disagreement-level data were not retained in a form that permitted retrospective reliability analysis. Inter-rater reliability could consequently not be calculated, and the consistency of initial assessments among individual SMEs could not be quantified. Consensus discussion may have supported agreement on final scores but does not provide evidence of independent rating reliability.

The study assessed bilingual and locally contextualised content but did not separately label or evaluate code-switching. Supplementary Table S1 presents Malay chatbot-generated dialogue alongside an English translation rather than alternation between languages within a single exchange. The framework's ability to interpret or reproduce natural Malay–English code-switching therefore remains unknown. Cultural and linguistic authenticity was also evaluated predominantly by clinicians rather than by patients, linguists, or intended learners.

The framework was limited to text-based Year 3 internal medicine scenarios. It could not reproduce or evaluate tone of voice, speech rate, pauses, facial expression, eye contact, gestures, body language, or other non-verbal components of clinical communication. Emotional expression conveyed through vocal tone or non-verbal behaviour was therefore neither incorporated into the training data nor evaluated. Consequently, the framework represents only part of the communication processes involved in authentic history-taking.

Interactions with real patients may also be substantially more complex and less structured than simulated interactions with an LLM. Patients experiencing pain, emotional distress, communication or language barriers, cognitive difficulties, or impaired recall may provide fragmented, ambiguous, inconsistent, or incomplete accounts rather than disclose information clearly and sequentially. Because an LLM may respond more coherently and predictably than patients encountered in clinical practice, repeated reliance on such simulations could create unrealistically high expectations regarding the ease of eliciting a clinical history. The framework should therefore be used as a preparatory and supplementary resource alongside supervised encounters with standardised and real patients, where learners can develop the flexibility required to manage uncertainty, distress, communication difficulties, and disorganised information.

The dataset captured clinician inquiry–AI-generated patient response pairs but did not model complete consultations through to a physician's diagnostic or management decision. No predefined consultation stopping rule was used, and the number of questions required before reaching a clinical decision was not recorded. The study therefore cannot determine how many questions a learner or physician would need to complete an encounter or reach a diagnosis.

The recurrent gaps identified by the SMEs—including compound questioning, poor sequencing, incomplete clinical probing, repetition, linguistic and terminological inaccuracies, over-explanation, and unrealistic patient responses—may affect the framework's educational authenticity and suitability for unsupervised use. These findings indicate that targeted refinement, continued expert review, and educator supervision are required before implementation.

The automated benchmark used 250 held-out patient profiles. Patient-profile-level partitioning prevented direct overlap of the held-out profiles with the fine-tuning partition; however, the holdout profiles originated from the same overall corpus-development process. The benchmark therefore represented an internal model-selection evaluation rather than an independently developed external test set. Generalisation to clinician-authored cases, other institutions, different patient populations, or learner-generated interactions was not assessed.

The benchmark relied on a single automated LLM judge without uncertainty estimates or blinded human adjudication across the four model configurations. In particular, clinicians did not independently evaluate the post-fine-tuning benchmark outputs, and no human–machine agreement analysis could therefore be performed. The study also did not undertake a targeted safety evaluation of the final fine-tuned model, including systematic measurement of medication errors, diagnostic inaccuracies, hallucinations, unsafe content, refusal behaviour, or failure of guardrails. The automated results should consequently be interpreted as preliminary internal model-development findings rather than evidence of clinical validation or readiness for unsupervised student-facing deployment.

Finally, dialogue generation was conducted through a conversational web interface rather than a programmatic API. Decoding parameters were therefore not configurable or recorded, no user-defined random seed was applied, and the underlying provider-managed model checkpoint may have changed during the collection period. Exact regeneration of the source corpus is consequently not possible. To improve methodological transparency, the prompt templates and scenario-parameter structure are provided in the Supplementary Material, while the substantive clinical evaluation in this study rests on the consultant-reviewed corpus rather than exact reproducibility of the initial generation process.

## Future directions

Future refinement should respond directly to the limitations identified by the SMEs. Compound questions and poor sequencing could be reduced through consultation-state tracking and instructions encouraging one focused question at a time. Incomplete probing could be addressed by mapping each encounter to a curriculum-aligned history-taking framework, while repetition could be reduced through dialogue-memory mechanisms that track information already requested or disclosed. When learners ask about information that is absent from the assigned patient profile, the model should request clarification of the question's purpose rather than generate unsupported clinical facts.

Simulated-patient realism could be strengthened through more consistent progressive disclosure, variable health-literacy levels, partial recall, hesitation, uncertainty, and context-dependent language. Overly formal Malay and inappropriate medical terminology should be replaced with authentic lay expressions validated by Malaysian clinicians, patients, linguists, medical educators, and learners. Because vocal tone and non-verbal behaviour were not represented in the present text-based dataset, future evaluation of emotional expression would require appropriately designed multimodal data.

Bilingual capability and code-switching should be evaluated as separate model capabilities. Future datasets should explicitly label within-utterance and between-turn language alternation and include naturally occurring patterns of Malay–English code-switching. Evaluation should also examine whether the model appropriately adapts its language to different levels of health literacy, cultural backgrounds, and clinical contexts.

Future corpus development should also extend beyond English and Malay to include Mandarin and other Chinese varieties, Tamil, and other languages relevant to Malaysian clinical practice. Demographic attributes should be defined prospectively rather than inferred retrospectively, enabling explicit evaluation of representational diversity, cultural appropriateness, and potential stereotyping or demographic bias.

Future studies should retain patient-profile, consultation-session, and evaluator identifiers and account for entries nested within consultations and reviewers. Independent ratings should be retained before consensus discussions to permit assessment of inter-rater reliability. Criterion-level scores should also be collected to support separate evaluation of clinical accuracy, clinical relevance, completeness, coherence, linguistic appropriateness, and conversational authenticity.

Future model comparisons should incorporate independently developed clinical test scenarios, paired response-level analyses, uncertainty estimates, and blinded evaluation of post-fine-tuning outputs by clinical educators, learners, and, where appropriate, patients. Human and automated evaluators should assess the same outputs to permit formal assessment of human–machine agreement. A dedicated clinical safety evaluation should quantify medication, diagnostic, factual, and other clinically consequential errors and examine model behaviour when faced with unsupported, ambiguous, or out-of-scope questions before student-facing implementation. Prospective multicentre studies should subsequently evaluate history-taking completeness, communication quality, diagnostic reasoning, learner confidence, knowledge retention, OSCE performance, and transfer to authentic patient encounters.

Multimodal development could incorporate speech, tone, facial expressions, and other non-verbal cues. Such development should be accompanied by evaluation of clinical safety, latency, privacy, bias, data governance, accessibility, and learner cognitive load before routine educational implementation.

## Conclusion

This study demonstrates the feasibility of developing a consultant-reviewed and culturally and linguistically adapted English–Malay clinical dialogue corpus and using it for parameter-efficient fine-tuning of an LLM for simulated clinical history-taking. Holistic SME ratings of the development corpus were generally favourable, while qualitative review identified important limitations involving questioning technique, clinical probing, terminology, linguistic authenticity, factual accuracy, and simulated-patient realism.

The LoRA fine-tuned model achieved the highest observed score in the internal automated benchmark; however, this comparison was intended for model development and selection and does not establish statistical, clinical, educational, or externally generalisable superiority. Post-fine-tuning outputs were not independently evaluated by clinicians, and targeted safety testing of the final model was not undertaken in the present study. The framework may provide a scalable and contextually relevant basis for further development of simulated history-taking tools, but blinded human evaluation, dedicated safety assessment, external validation, formal OSCE alignment, and prospective learner-outcome studies are required before routine student-facing implementation.

## Data Availability

The raw data supporting the conclusions of this article will be made available by the authors, without undue reservation.
