# Supplementary material for: Development and expert evaluation of a culturally adapted large language model for Malay clinical history-taking simulation: mixed methods study
Source: Front Med (Lausanne). 2026 Sep 30;13:1895321. doi: 10.3389/fmed.2026.1895321 (PMC13661435; doi:10.3389/fmed.2026.1895321)
Supplement: Supplementary file 1 [file Table1.docx]

**Supplementary Methods S1.** AI-Assisted Corpus Generation and Prompt Documentation

| **Parameter** | **Recorded information** |
| --- | --- |
| Provider and model | OpenAI GPT-5 |
| Interface | ChatGPT web interface; not the OpenAI API |
| Access date used in the manuscript | 7 August 2025 |
| Corpus size | 63,250 paired dialogue entries across nine internal medicine systems |
| User-configurable decoding parameters | None recorded; provider-managed defaults applied |
| Data transfer | Manual transfer to the study dataset repository |

## S1.1 Virtual-Patient System Prompt Used in Fine-Tuning and Inference

The following role-conditioning template was recovered verbatim from the executed fine-tuning notebook. Bracketed field names indicate the scenario-specific values inserted for each simulated patient.

ANDA ADALAH PESAKIT. ANDA BUKAN DOKTOR.

KLINIK: <clinic>
PROFIL PESAKIT:
<patient profile>
Diagnosis klinikal: <clinical diagnosis>
Topik perubatan: <medical topic>

Peraturan ringkas:
- Jawab sebagai PESAKIT sahaja, bukan doktor.
- Guna 'saya' untuk diri sendiri dan 'doktor' untuk pelajar.
- Jawapan dalam Bahasa Malaysia harian.
- Elakkan istilah medik yang rumit bila bercakap dengan doktor.

## S1.2 Scenario Parameters

| **Field** | **Function** |
| --- | --- |
| Clinic | Identified the relevant internal medicine clinical system. |
| Patient profile | Specified demographic, occupational, social, health-literacy, emotional, and clinical characteristics. |
| Clinical diagnosis | Defined the assigned clinical condition and informational boundaries of the scenario. |
| Medical topic | Linked the simulated encounter to the relevant curriculum-aligned teaching topic. |
| Dialogue history | Provided preceding clinician and patient turns during inference so that the model could generate the final patient response in context. |
