# Supplementary material for: Development and expert evaluation of a culturally adapted large language model for Malay clinical history-taking simulation: mixed methods study
Source: Front Med (Lausanne). 2026 Sep 30;13:1895321. doi: 10.3389/fmed.2026.1895321 (PMC13661435; doi:10.3389/fmed.2026.1895321)
Supplement: Supplementary file 2 [file Table2.docx]

**Supplementary Methods S2.** Internal Model Benchmarking and Automated Evaluation

## S2.1 Automated Judge

| **Parameter** | **Recorded value** |
| --- | --- |
| Judge model | Google Gemini 2.5 Flash |
| Access route | OpenRouter; model identifier google/gemini-2.5-flash |
| Access date | Not retained in the available technical log |
| Prompting | Few-shot; 120 SME-scored exemplars, comprising 24 examples for each score from 1 to 5 |
| Exemplar source | Training partition only; no validation or held-out test profiles |
| Output instruction | Return only one Patient Response Score without reasoning |
| Temperature | Not set in the request; passed through to the upstream provider default |
| Top-p and top-k | Not set in the request; passed through to the upstream provider defaults |
| Maximum output tokens | Not explicitly set in the available judge request record |
| Reasoning | Enabled through OpenRouter with requested budget_tokens = 10,000 |

## S2.2 Per-Item Judge Input Structure

{
 "Clinic": "...",
 "Medical Topic": "...",
 "Clinical Diagnosis": "...",
 "Role": "...",
 "Doctor Question": "...",
 "Patient Response": "...",
 "Patient Response Score": null
}
