# Supplementary material for: Development and expert evaluation of a culturally adapted large language model for Malay clinical history-taking simulation: mixed methods study
Source: Front Med (Lausanne). 2026 Sep 30;13:1895321. doi: 10.3389/fmed.2026.1895321 (PMC13661435; doi:10.3389/fmed.2026.1895321)
Supplement: Supplementary file 3 [file Table3.docx]

**Supplementary Table S1.** Example Malay Simulated Dialogue With English Translation

| **Role** | **Original Malay dialogue** | **English translation** |
| --- | --- | --- |
| Doctor | Selamat pagi Encik, saya Dr. Nadia. Boleh saya tahu nama penuh dan umur encik? | Good morning, sir. I am Dr Nadia. May I know your full name and age? |
| Patient | Saya Faizul bin Mohamad, umur saya 42 tahun doktor. | I am Faizul bin Mohamad, and I am 42 years old, doctor. |
| Doctor | Apa yang membawa encik ke klinik hari ini? | What brings you to the clinic today? |
| Patient | Saya asyik sakit di bahagian rusuk belakang, dan air kencing kadang berdarah. Dah dua kali jadi macam ni. | I keep having pain around the back of my flank, and sometimes there is blood in my urine. This has happened twice. |
| Doctor | Encik sakit di bahagian mana? Tajam atau berdenyut? | Where exactly is the pain? Is it sharp or throbbing? |
| Patient | Sakit di belakang kanan, rasa tajam dan datang secara tiba-tiba. | The pain is on the right side of my back. It feels sharp and comes on suddenly. |
