# Supplementary material for: Development and expert evaluation of a culturally adapted large language model for Malay clinical history-taking simulation: mixed methods study
Source: Front Med (Lausanne). 2026 Sep 30;13:1895321. doi: 10.3389/fmed.2026.1895321 (PMC13661435; doi:10.3389/fmed.2026.1895321)
Supplement: Supplementary file 4 [file Table4.docx]

**Supplementary Table S2.** Retained-Score Distribution

| **Retained score** | **Doctor inquiry, n (%)** | **Patient response, n (%)** |
| --- | --- | --- |
| 3 | 1,824 (3.22%) | 1,194 (2.11%) |
| 4 | 16,767 (29.62%) | 15,420 (27.24%) |
| 5 | 38,017 (67.16%) | 39,994 (70.65%) |
| Total retained ratings | 56,608 (100.00%) | 56,608 (100.00%) |
