# Supplementary material for: Development and expert evaluation of a culturally adapted large language model for Malay clinical history-taking simulation: mixed methods study
Source: Front Med (Lausanne). 2026 Sep 30;13:1895321. doi: 10.3389/fmed.2026.1895321 (PMC13661435; doi:10.3389/fmed.2026.1895321)
Supplement: Supplementary file 5 [file Table5.docx]

**Supplementary Table S3.** Complete Fine-Tuning Configuration

| **Parameter** | **Recorded value** |
| --- | --- |
| Base checkpoint | mesolitica/Malaysian-Llama-3.1-8B-Instruct-v0.1 |
| Model size | 8,072,204,288 parameters |
| Hardware used for training | 1 x NVIDIA Tesla T4; 14.7 GB reported memory |
| Training wall-clock time | Approximately 2 hours 14 minutes |
| Software | Unsloth 2025.11.6; Transformers 4.56.2; TRL 0.22.2; PyTorch 2.9.1+cu128; Python 3.11.13 |
| Quantisation | 4-bit QLoRA using bitsandbytes |
| Computation type | FP16; bfloat16 unsupported on the Tesla T4 runtime |
| Maximum sequence length | 2,048 tokens |
| Chat template | Llama 3.1 template through Unsloth get_chat_template |
| LoRA rank | 16 |
| LoRA alpha | 16 |
| LoRA dropout | 0 |
| LoRA bias | None |
| Target modules | q_proj, k_proj, v_proj, o_proj, gate_proj, up_proj, and down_proj |
| Trainable parameters | 41,943,040 (0.52% of all parameters) |
| Gradient checkpointing | Unsloth gradient checkpointing enabled |
| Optimiser | adamw_8bit |
| Weight decay | 0.01 |
| Learning rate | 2 x 10^-4 |
| Learning-rate schedule | Linear |
| Warm-up steps | 5 |
| Epochs | 1 |
| Optimiser steps | 63 |
| Per-device training batch size | Runtime summary: 8; notebook TrainingArguments source: 4 |
| Gradient-accumulation steps | 4 |
| Effective training batch size | Runtime summary: 32 (8 x 4 x 1 GPU) |
| Per-device evaluation batch size | 1 |
| Evaluation-accumulation steps | 4 |
| Packing | False |
| Loss masking | Loss calculated on assistant or simulated-patient turns only; clinician turns masked |
| Training seed | 3407 |
| Data-split seed | 42 |
| Evaluation frequency | Every 30 optimiser steps |
| Checkpoint frequency | Every 30 optimiser steps |
| Early stopping | Not applied |
| Checkpoint selection | Checkpoint with the lowest observed validation loss restored after training |
| Reported validation loss | Step 30: 1.0602; step 60: 0.9840 |
| Reported training loss | Step 30: 1.0424; step 60: 1.0161 |
| Output quantisations | GGUF q4_k_m and q8_0 |
